# Dynamic Cipher Puzzle for Efficient Broadcast Authentication in Wireless Sensor Networks

**DOI:** 10.3390/s18114021

**Published:** 2018-11-18

**Authors:** Farah Afianti, Wirawan, Titiek Suryani

**Affiliations:** Department of Electrical Engineering, Faculty of Electrical Technology, Institut Teknologi Sepuluh Nopember, Jalan Raya ITS, Keputih, Sukolilo, Surabaya 60111, Indonesia; wirawan@ee.its.ac.id (W.); titiks@ee.its.ac.id (T.S.)

**Keywords:** Dynamic Cipher Puzzle, broadcast authentication, signature based DoS

## Abstract

The use of signature-based broadcast authentication for code and data dissemination in wireless sensor networks (WSNs) cannot be avoided. It increases security but requires high computation. Adversaries can exploit the latter condition as an opportunity to send many false signatures. Filtering methods can overcome this vulnerability. Cipher Puzzle is a filtering method that has low storage overhead along with high security, especially against denial of service (DoS) attacks. However, its number of hash iterations cannot be bounded, which causes sender-side delay. This paper proposes a Dynamic Cipher Puzzle (DCP), which uses a threshold function to limit the number of hash iterations. Hence, time at the sender-side can be used more efficiently. Besides, its dynamic puzzle-strength increases the obscurity of the transmitted packet. Simulation and experimental results were analyzed with Arduino 2560. The theoretical results show that the quadratic function outperformed the compared methods. The scheme decreased sender-side delay by 94.6% with a guarantee of zero solution probability in 1.728 $\times 10^{-13}$. The experimental results show that the consumption of resources at the sensor node increases with an acceptable value. Moreover, DCP increases the complexity for the attacker to implement probability and signature-based DoS attacks.

## 1. Introduction

Wireless sensor networks (WSNs) connect users to many sensor nodes in a remote area. Users, especially network administrators, have to perform maintenance on these sensor nodes, which are usually located in places that are hard to reach. An administrator will need to send a patching program, code to fix bugs, or add functions, commands, confidential data, etc. to all of the sensor nodes. Broadcast or multicast is an efficient way for the administrator to communicate with the sensor nodes. However, broadcast communication is vulnerable against flooding with false messages [1,2].

Sensor nodes can be protected from attacks using an authentication mechanism that prevents illegitimate users from broadcasting fake messages. There are two types of broadcast authentication, i.e., Message Authentication Code (MAC) and Digital Signature. They are distinguished based on their cryptographic mechanism [3]. The utilization of MAC in WSNs has been discussed in [4,5,6,7]. Digital signature has also been successfully implemented in WSN environments, employing elliptic curve variants [8,9,10] or RSA [11]. Unfortunately, both cryptographic mechanisms are weak against DoS attacks. This can deplete the energy of the sensor nodes so that they can no longer be accessed. For Digital Signature, this effect is stronger because a Digital Signature needs more computation than MAC. Attackers can use this vulnerability to send many false digital signatures. From an adversary’s point of view, this mechanism is more effective than injecting radio frequency noise [12]. In view of this, filtering methods have been developed. They are used as a complement of the main signature, not a replacement. Their computation must be lower than that of the main signature so that the sensor node can decide as soon as possible whether a packet is fake.

Cipher Puzzle [13] is a filtering method that has low storage overhead along with high security, especially against DoS attacks. Furthermore, it was the first filtering scheme to aggregate confidentiality and resistance to DoS attacks in WSNs. However, its number of hash iterations cannot be bounded which causes sender-side delay. Hence, a mechanism to limit this delay is needed. This paper has two main contributions. Firstly, a new method called Dynamic Cipher Puzzle is proposed, which uses a threshold function to decrease sender-side delay. It generates the puzzle strength dynamically for each puzzle solution produced. Even though it has a low number of hash iterations, it has a guarantee of zero solution probability in 1.728 $\times 10^{-13}$. Secondly, the obscurity of the transmitted packet is increased by using a tagging mechanism. Instead of sending the puzzle strength value implicitly, a tag is used. The adversary has to guess the value of the puzzle strength for each message exchange which is more difficult than guessing a static puzzle strength value that is stored in the sensor node. Furthermore, experimental results from a test-bed implementation using Arduino Mega 2560 are presented in this paper to analyze the proposed filtering method’s performance at the sensor node.

The rest of this paper is organized as follows. The notation used in this paper is summarized in Table 1. Section 2 gives a brief overview of previous works that incorporate several variant of puzzle mechanisms. Section 3 explains the material and methods of the experiment. Section 4 explains the design and implementation of the proposed method. In Section 5, the result of the experiment is discussed. Section 6 contains a brief explanation of the security analysis. Section 7 provides a summary of this paper and possible topics for future work.

## 2. Related Works

Filtering methods or weak authenticators are important, especially for protecting wireless sensor networks against DoS attacks. These attacks exploit the high computation of signature verification at the receiver-side. The Client Puzzle scheme was introduced to protect the resources of the server [14]. The main objective of this filtering method is resource efficiency. The puzzle scheme has been modified so it can be implemented in WSN environments. Message Specific Puzzle (MSP) was introduced as the first puzzle scheme for resource-constraint devices [15]. It utilizes a hash function to produce a puzzle for the required security strength and a keychain for the session key. This method has low complexity but requires a powerful sender. To avoid using a puzzle, multiple keychains have been built to differentiate every sender’s keychain [16]. This can decrease sender-side delay but every sensor node must store the key commitment from each other sender which increases storage overhead in the sensor node. In other studies, commitment values were utilized instead of a puzzle [12,17], while implementing the same session key mechanism. This requirement becomes stricter when a secret message needs to be sent to the WSN. Cipher Puzzle adds the encrypt-then-MAC (ETM) mechanism to ensure confidentiality. Cipher Puzzle has high security but— similar to MSP— also induces sender-side delay. It has been stated that, in MSP, a 22-bit puzzle strength is secure with an acceptable delay [15]. There is no information for suitable puzzle strength in Cipher Puzzle, but it has been stated that the value is a multiplication of eight bits, i.e., it can be 8, 16, 24, and so on. The mean value of the number of hash iterations in every puzzle generation is $2^{L}$ [16]. Consequently, if Cipher Puzzle uses puzzle strength of 3 bytes, or 24 bits, then the delay is about $2^{24}$, which is the average time needed by the sender to find the puzzle’s solution. If each iteration costs about 0.1 ms (using an Intel Core i3-2310M CPU @ 2.10GHz - 4CPU processor and 3087 MB DDR3) then the time needed for sending each packet is about $2^{24}\times 0.1$ ms = 1,677,721.6 ms, or 27.96 min. This is unreasonable. Moreover, the puzzle strength is fixed and set in the initialization phase. Hence, the time needed to send a message varies and cannot be known; the sender can neither control nor predict it. Dynamic Message Puzzle (DMP) has been proposed in [18], which utilizes dynamic puzzle length in MSP. This scheme is approximated using heuristic methods that can still be optimized to reduce the delay at the receiver-side. Besides, the messages are sent as plaintext, which is not suitable for code dissemination.

The three mentioned previous puzzle schemes, Client Puzzle, Message Specific Puzzle and Cipher Puzzle, have the same objective. The differences between them are described in Table 2.

## 3. Cipher Puzzle Overview

Cipher Puzzle is a reconstruction of MSP by adding a confidential security property so it can be implemented for code dissemination. It is a rising approach that has attracted much attention. The objective is to transfer code, confidential data or commands (data dissemination) from the network administrator to the sensor nodes. As a result, the administrator can maintain the sensor nodes remotely. For example, in mobile electronic nose application, the recent procedures must be transmitted to the sensor nodes continuously [19]. It can be done in a secure way without accessing the physical sensor nodes. Cipher Puzzle is implemented in the Deluge protocol and has three agreement mechanisms, i.e., advertise, request, and update verification. Cipher Puzzle is used as the first line of defense in the advertisement packet. Consequently, the system is robust against signature- and request-based DoS attacks.

**Figure 1:**
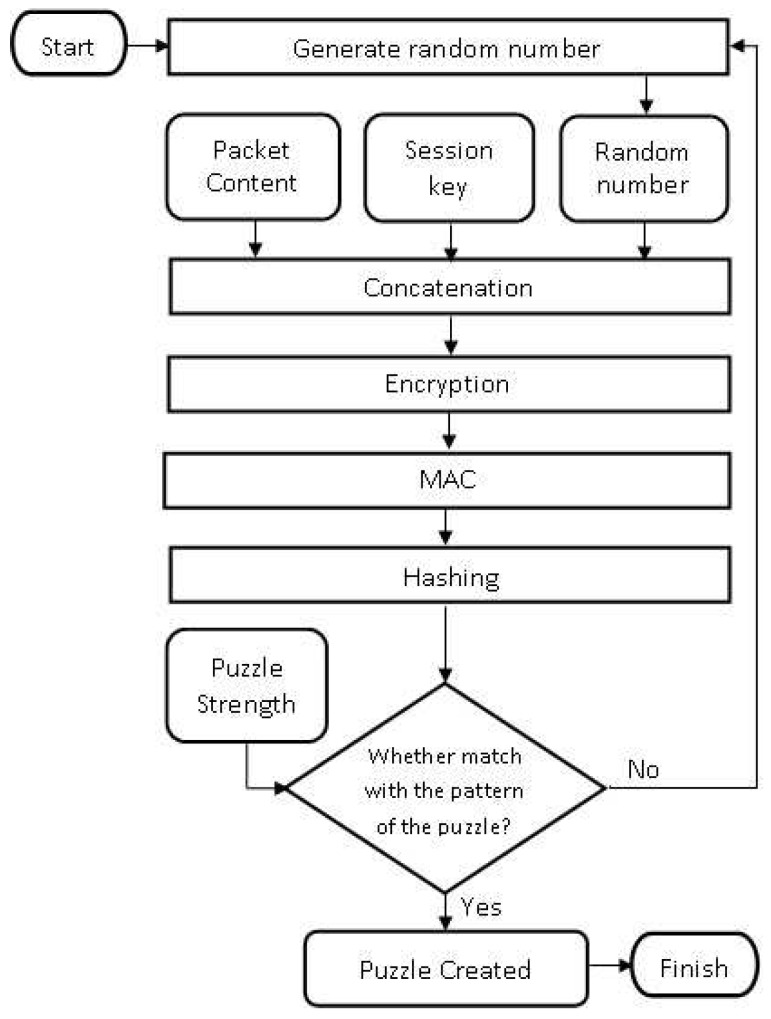
Cipher Puzzle constructor.

The detailed steps of Cipher Puzzle Constructor can be seen in Figure 1. It starts with session key generation using a one-way key chain (see Section 3.1 for details). Then, concatenation of the packet content, the session key, and a random number is done. The third step is ETM. The result is hashed and then the first *L* bits are split. The output is compared with the pattern that comes from the hash result of the digital signature (Section 3.2). The details of the digital signature are explained in Section 3.3.

### 3.1. One-Way Key Chain

The session key for each packet is calculated using a one-way key chain mechanism. The idea for the key agreement comes from [20]. It aims to deny illegitimate packets from attackers who try to generate fake puzzle solutions [15]. Before executing the next process, the session key will be checked so if it is not appropriate the packet will be dropped. The drawback of this key generation process is the finite number of keys that can be produced [21]; this parameter must be defined in the initialization phase.

The main algorithm in the one-way keychain is the hash function. The first step is random number generation. This value is placed in the last position. The key generation is done by Equation (1) [16]:

(1) $$K_{i}=F_{H}\left(K_{i+1}\right)$$

where $K_{i}$ is the *i*th session key.

This process is repeated until the number of expected $\left(N_{key}\right)$ keys is reached. The set of keys that are used as session keys is represented by Equation (2):

(2) $$\overset{N_{key-1}}{\underset{i=1}{⋃}}F_{H}^{i}\left(x\right)⋃x;x\in random$$

where $F_{H}^{i}\left(x\right)$ is the hash function of value *x*, which is repeated *i* times.

The first key is called the commitment key. This value is sent to the receiver. It is used in the verification process.

### 3.2. Puzzle Solution

Puzzle solution construction is the last step before the packet is sent to the receiver. The idea is to find the hash function result that matches a specific pattern, called the Cipher Puzzle pattern. It starts with concatenation to aggregate several sent parameters, including a random number as puzzle solution and then hashes them. Subsequently, the result is compared with the pattern. The Cipher Puzzle pattern consists of the first *L* bits of the hash result of the digital signature from the packet content, as expressed in Equation (3):

(3) $$ETM_{K_{0}}\left(M∥K_{idx}∥P_{idx}\right)=F_{H}\left(Sig\left(M\right)\right)$$

where $ETM_{K_{0}}\left(x\right)$ is the encrypt-then-MAC of *x* using session key $K_{0}$.

If it fails, the process will generate another random number as puzzle solution candidate, $P_{idx}$. Otherwise, the sender can send the packet.

### 3.3. Digital Signature

Signature generation is computationally expensive, especially for resource-constrained devices. Today, this limitation can be overcome; there are several implementations of public key cryptography (PKC) based authentication methods for resource-constrained environments [8,22,23,24]. However, the number of PKC-based authentications that can be executed in the sensor node is limited.

Among the existing digital signatures, the Elliptic-Curve Cryptography (ECC) variants [25] were selected for Cipher Puzzle and MSP. Cipher Puzzle uses TinyECC [8] because the system is implemented in TinyOS, while the Elliptic Curve Digital Signature Algorithm (ECDSA) was selected for MSP [15]. This PKC is the most appropriate algorithm for WSN environments because of its key length [26]. It only consists of 40 bytes for a 40-byte signature which is smaller than RSA, which needs 128 bytes for the same security level [10].

## 4. Dynamic Cipher Puzzle (DCP)

**Figure 2:**
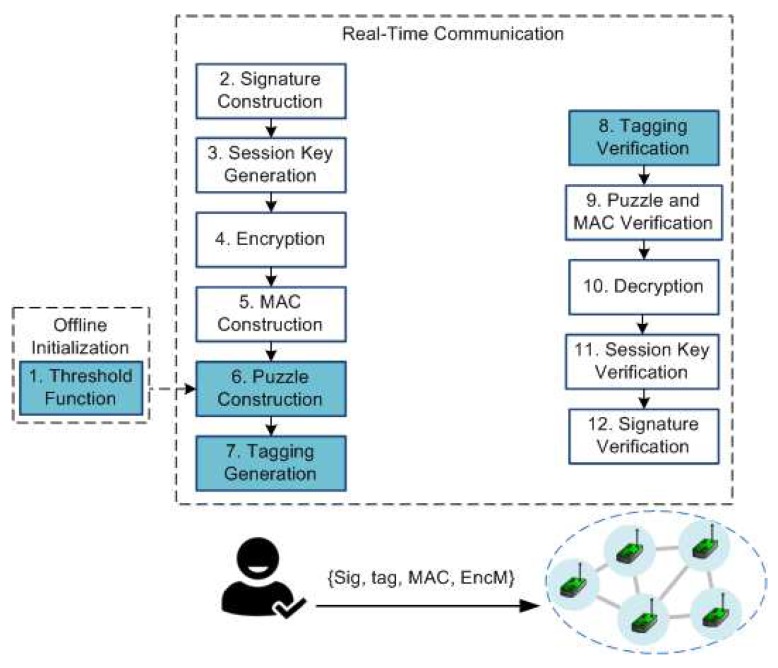
Dynamic Cipher Puzzle. The blue rectangles indicate the proposed methods.

A block diagram of the proposed system is shown in Figure 2. The system starts with offline initialization. This is explained in Section 4.1, followed by real-time communication in Section 4.2.

### 4.1. Offline Initialization

This step aims to produce the best threshold function, appropriate for the characteristics of Cipher Puzzle. We need information about how many trials are needed to fulfill any given probability of finding the puzzle solution. It is stated in [13,15] that the probability of finding a solution in the puzzle scheme can be derived from binomial trial. Therefore, the number of trials needed for a given $P_{success}$ and puzzle strength *L* is expressed by Equation (4).

(4) $$\begin{array}{cc} P_{success} & =1-{\left\{1-{\left(\frac{1}{2}\right)}^{L}\right\}}^{n}\\ {\left\{1-{\left(\frac{1}{2}\right)}^{L}\right\}}^{n} & =1-P_{success}\\ \log_{10}{\left\{1-{\left(\frac{1}{2}\right)}^{L}\right\}}^{n} & =\log_{10}\left\{1-P_{success}\right\}\\ n\log_{10}\left\{1-{\left(\frac{1}{2}\right)}^{L}\right\} & =\log_{10}\left\{1-P_{success}\right\}\\ n & =\frac{\log_{10}\left\{1-P_{success}\right\}}{\log_{10}\left\{1-{\left(\frac{1}{2}\right)}^{L}\right\}} \end{array}$$

where *n* is the number of hash iterations.

In this paper, we propose dynamic puzzle strength (*L*), ranging from $L_{max}$ to 1. Thus, the maximum number of hash iterations for each trial in DCP is expressed in Equation (5).

(5) $$n_{DCP}=\sum_{i=0}^{L_{max}-1}\frac{\log_{10}\left\{1-P_{\left[success,L_{max}-i\right]}\right\}}{\log_{10}\left\{1-{\left(\frac{1}{2}\right)}^{\left(L_{max}-i\right)}\right\}}$$

The rate of success probability for each puzzle strength also determines the zero solution probability of the system. Furthermore, the proposed method incorporates the puzzle strength dynamically and the success probability of each puzzle strength affects the success probability for the overall system. This case can be approached by using set theory. The universal class (universal set) is the class of all sets [27]. In this case, the universal set of system probability is the union of the success and failure probability. It is computed by Equation (6).

(6) $$\begin{array}{cc} S & =P_{success}\cup \bar{P_{success}}\\ S & =P_{success}\cup P_{failure} \end{array}$$

**Figure 3:**
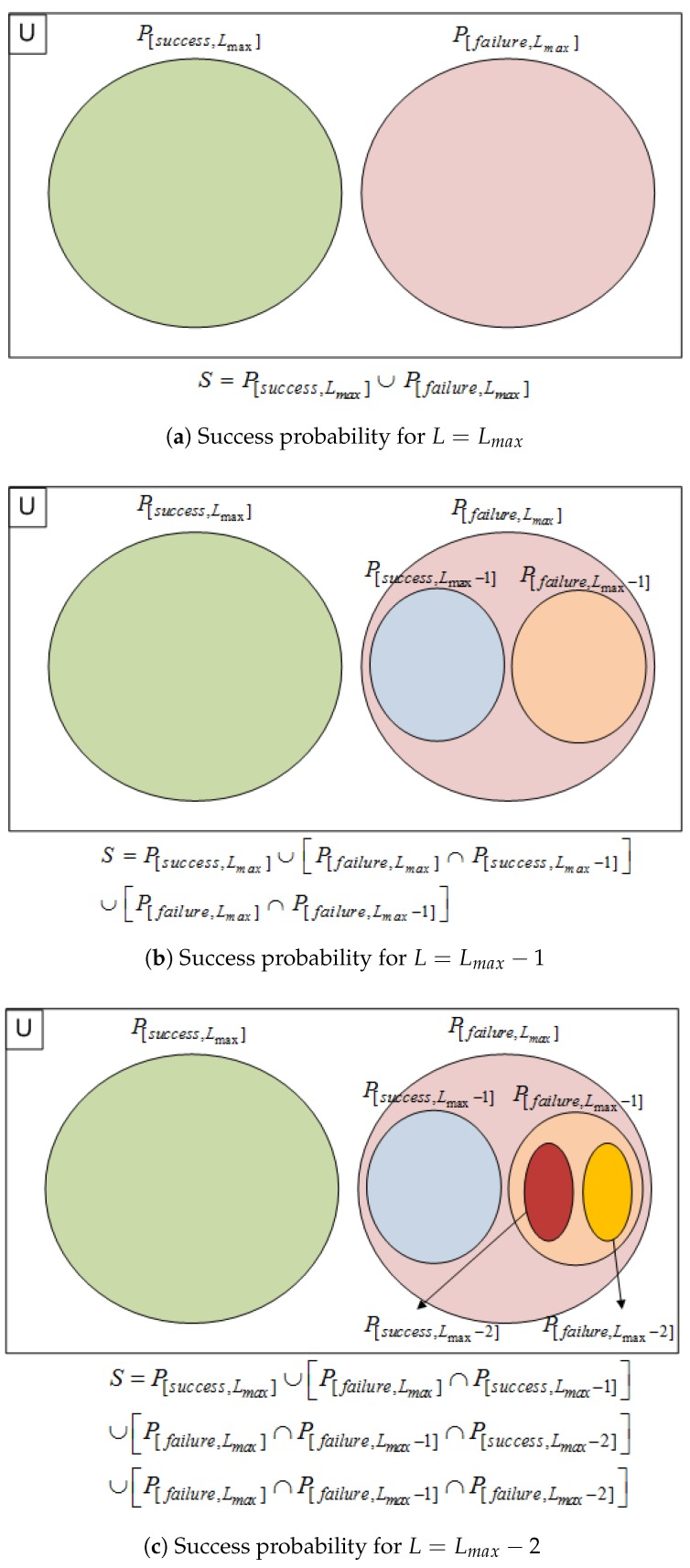
Venn diagram of DCP’s success probability.

The starting puzzle strength position for DCP trial is at $L_{max}$ and the result is divided into two conditions, i.e., “found” or “failed”. In the case of “failed”, the system lowers the puzzle strength and starts another trial with a lower puzzle strength and another success probability. This cycle stops when the puzzle is found or the puzzle strength is zero, i.e., zero solution. This condition is illustrated by the Venn diagram in Figure 3. It shows the first three universal sets from $L_{max}$ to $\left(L_{max}-2\right)$. Iteration continues until the puzzle-strength value is 1. Then, the overall system probability is formulated by Equation (7).

(7) $$\begin{array}{cc} S & =\overset{L_{max}}{\underset{i=1}{⋃}}PS_{\left[success,i\right]}\cup \overset{L_{max}}{\underset{i=1}{⋂}}P_{\left[failure,i\right]}\\ PS_{\left[success,i\right]} & =\left\{\begin{array}{cc} P_{\left[success,i\right]} & i=L_{max}\\ P_{\left[success,i\right]}{⋂}_{j=1}^{n=L_{max}-i}P_{\left[failure,i+j\right]} & i<L_{max} \end{array}\right. \end{array}$$

where *S* denotes the probability of the system, PS describes the success probability for each stated puzzle strength and *i* is the puzzle strength.

The best threshold function in DCP is the function with the lowest value of zero solution probability, as shown in Equation (8).

(8) $$\begin{array}{cc} P_{ZS} & =\overset{L_{max}}{\underset{i=1}{⋂}}P_{\left[failure,i\right]}\\ & =\overset{L_{max}}{\underset{i=1}{⋂}}\left\{1-P_{\left[success,i\right]}\right\} \end{array}$$

In addition, the other considered parameters in determining the best threshold function are the mean of number of hash iterations and its variance. The mean number of hash iterations is important to know because it shows the delay at the sender-side, so that the sender can estimate the time needed to send the message. It is measured by repeatedly using a threshold function in real-time communication. In this paper, the experiment was repeated 60 times to fulfill a small sample. The last parameter calculated is the standard deviation, using Equation (9).

(9) $$\begin{array}{cc} \sigma_{L} & =\sqrt{E\left(L^{2}\right)-{\left[E(L)\right]}^{2}}\\ & =\sqrt{\left[\sum_{i=0}^{L_{max}}\left(L_{i}^{2}.PS_{\left[success,i\right]}\right)\right]-{\left[\sum_{i=0}^{L_{max}}\left(L_{i}.PS_{\left[success,i\right]}\right)\right]}^{2}} \end{array}$$

The dynamic puzzle strength is treated as a discrete random variable. Variance measurement aims to know the diversity level of puzzle strength. It shows the spread of the puzzle strengths in relation to their mean. The value is high if the data are spread widely in relation to their mean and low if they are clustered near the mean. A higher variance indicates higher complexity to guess the sent puzzle strength.

**Figure 4:**
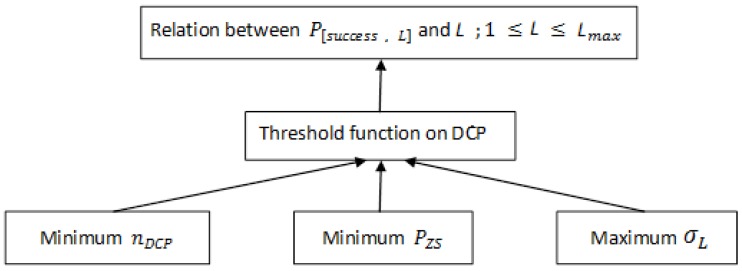
Parameters for selecting the best threshold function.

Based on Equation (4), the total number of hash iterations depends on the success probability for each puzzle strength ($P_{\left[success,L\right]}$). If the puzzle strength value is dynamic, then its success probability becomes dynamic too. Therefore, we build a relation between them based on the optimum parameters for selecting the best threshold functions, as illustrated in Figure 4.

**Figure 5:**
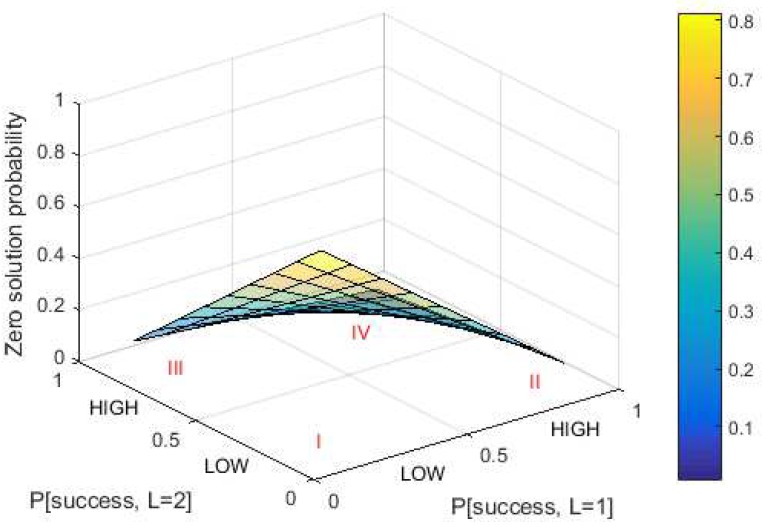
Zero solution probability in DCP for $L_{max}=2$.

The first parameter is the zero solution probability as expressed in Equation (8). DCP aims to get the lowest zero solution probability to makes sure that there will be a solution for each puzzle generation. The surface plot for the zero solution probability for maximum puzzle strength equal to 2 is shown in Figure 5. It can be seen that a higher value of puzzle strength and its success probability leads to a lower probability of zero solution.

**Figure 6:**
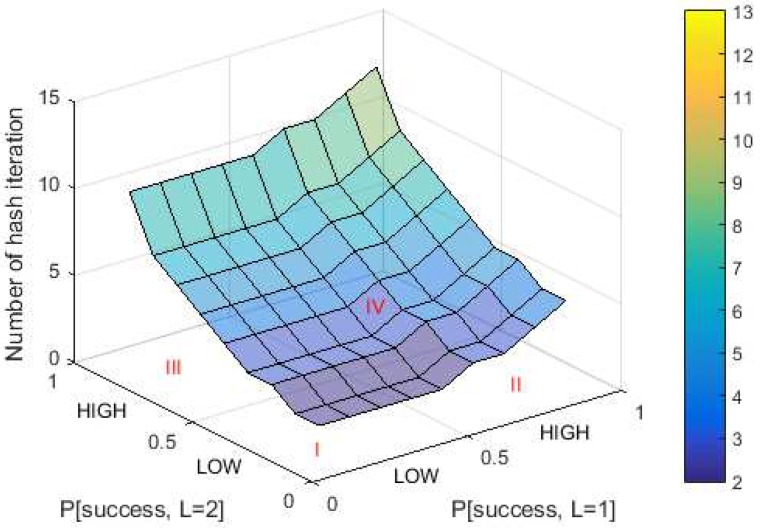
Number of hash iterations in DCP for $L_{max}=2$.

The second parameter is the number of hash iterations. The aim of this study was to decrease sender-side delay, which is indicated by a lower number of hash iterations for each puzzle generation. The surface plot for the number of hash iterations at maximum puzzle strength equal to 2 is shown in Figure 6. The lower the puzzle strength and its success probability, the lower the number of hash iterations.

**Figure 7:**
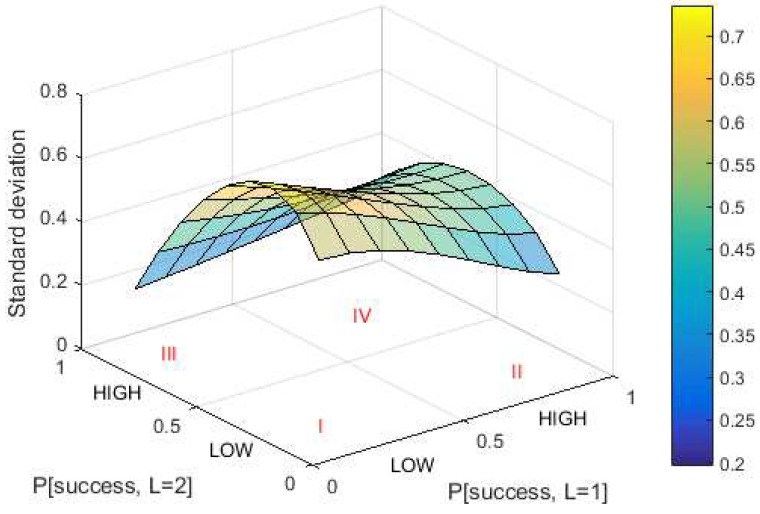
Standard deviation in DCP for $L_{max}=2$.

The third parameter is the standard deviation. DCP aims for a high standard deviation, which means high data diversity. A surface diagram of DCP’s standard deviation for $L_{max}$ equal to 2 is shown in Figure 7.

The area of success probability is divided into two areas to make it easier to analyze: “LOW” and “HIGH”. Low probability means the success probability is between 0 and 0.5, while high probability means the success probability is between 0.5 and 1. The optimum condition for success probability in DCP for $L_{max}$ = 2 is summarized in Table 3.

In Area I, the optimum value is reached by hash iteration ($n_{DCP}$) and standard deviation ($\sigma_{L}$), but the probability of zero solution ($P_{ZS}$) has the worst value of all areas. Conversely, in Area IV, the optimum value is reached by the probability of zero solution, while the worst value comes from the hash iteration and standard deviation. This indicates that a static value of success probability for each puzzle strength is not recommended. This is because if the success probability value is “LOW” then the zero solution probability is worst. In contrast, if the value of success probability is “HIGH” then the number of hash iterations and standard deviation is worst. The options remain are Areas II and III. The values of $n_{DCP}$ and $\sigma_{L}$ in Area II are better than in Area III. In Area II, the mean number of hash iterations is lower and the standard deviation are higher than in Area III. Meanwhile, the probability of zero solution is almost the same in both areas. Based on this analysis, Area II was selected as the basis for the relationship between success probability and puzzle strength. Area II reflects that a higher value of puzzle strength leads to a lower success probability. This hypothesis is supported by another study [18]. Based on the result of this paper, the value of success probability in the optimum threshold function comes from either a linear function that has a positive slope or from a concave function. Consequently, the success probability is built from both functions.

First, two coordinate points that are passed by a linear and quadratic equation are defined. The *x*-coordinates represent the puzzle-strength value in bits. The *y*-coordinates represent the success probability of each puzzle strength, ranging from zero to one. The first coordinate point ($x_{1},y_{1}$) is $\left(L_{max}+1,0\right)$. This means that it is impossible to have puzzle strength larger than $L_{max}$. The second point ($x_{2},y_{2}$) is (1,ε), where 0<ε<1. This is used as a performance parameter that reflects the expected success probability in the lowest puzzle-strength. A higher value of ε leads to a higher possibility of producing a solution at the lowest puzzle strength (L=1). A value close to 1 needs to be assigned to this variable. This paper expects $\varepsilon =1-10^{-10}$ as a representative value. Based on these points, a linear equation from the basic form is built as expressed in Equation (10).

(10) $$\begin{array}{cc} \frac{y-y_{1}}{y_{2}-y_{1}} & =\frac{x-x_{1}}{x_{2}-x_{1}}\\ \frac{P_{\left[success,L\right]}-P_{\left[success,L_{max}+1\right]}}{P_{\left[success,L_{1}\right]}-P_{\left[success,L_{max}+1\right]}} & =\frac{L-(L_{max}+1)}{L_{1}-\left(L_{max}+1\right)}\\ \frac{P_{\left[success,L\right]}-0}{(1-10^{-10})-0} & =\frac{L-(L_{max}+1)}{1-(L_{max}+1)}\\ P_{\left[success,L\right]} & =\left(1-10^{-10}\right)\frac{\left[L-\left(L_{max}+1\right)\right]}{\left[-L_{max}\right]} \end{array}$$

Furthermore, a quadratic equation formed by the success probability is derived from the basic quadratic function that passes point $\left(x_{1},0\right)$, as expressed in Equation (11).

(11) $$\begin{array}{cc} y & =a{\left(x-x_{1}\right)}^{2}\\ P_{\left[success,L\right]} & =a{\left(L-\left(L_{max}+1\right)\right)}^{2}\\ (L_{1},P_{\left[success,L_{1}\right]})\to & \;a=\frac{P_{\left[success,L_{1}\right]}}{{\left(L_{1}-\left(L_{max}+1\right)\right)}^{2}}\\ & \;a=\frac{\left(1-10^{-10}\right)}{{\left(1-\left(L_{max}+1\right)\right)}^{2}}\\ & \;a=\frac{\left(1-10^{-10}\right)}{{L_{max}}^{2}}\\ P_{\left[success,L\right]} & =\frac{\left(1-10^{-10}\right)}{{L_{max}}^{2}}{\left(L-\left(L_{max}+1\right)\right)}^{2} \end{array}$$

Both success probability Equations (Equations (10) and (11)), are combined with Equation (4) to get information about the number of trials needed in DCP for a high value of success probability. The first threshold function, using linear success probability, is defined by Equation (12).

(12) $$n=\frac{\log_{10}\left\{1-\left[\left(1-10^{-10}\right)\frac{\left[L-\left(L_{max}+1\right)\right]}{\left[-L_{max}\right]}\right]\right\}}{\log_{10}\left\{1-{\left(\frac{1}{2}\right)}^{L}\right\}}$$

The second threshold function, using quadratic success probability, is defined by Equation (13).

(13) $$n=\frac{\log_{10}\left\{1-\left[\frac{\left(1-10^{-10}\right)}{{L_{max}}^{2}}{\left(L-\left(L_{max}+1\right)\right)}^{2}\right]\right\}}{\log_{10}\left\{1-{\left(\frac{1}{2}\right)}^{L}\right\}}$$

### 4.2. Real-Time Communication

The next process after getting the threshold function is real-time communication implementation between sender, or administrator, and receiver, or sensor node. The detailed activities of the sender are explained by Algorithm 1, while Algorithm 2 gives the detailed activities on the receiver side.

**Algorithm 1:** Dynamic Cipher Puzzle constructor at the sender-side

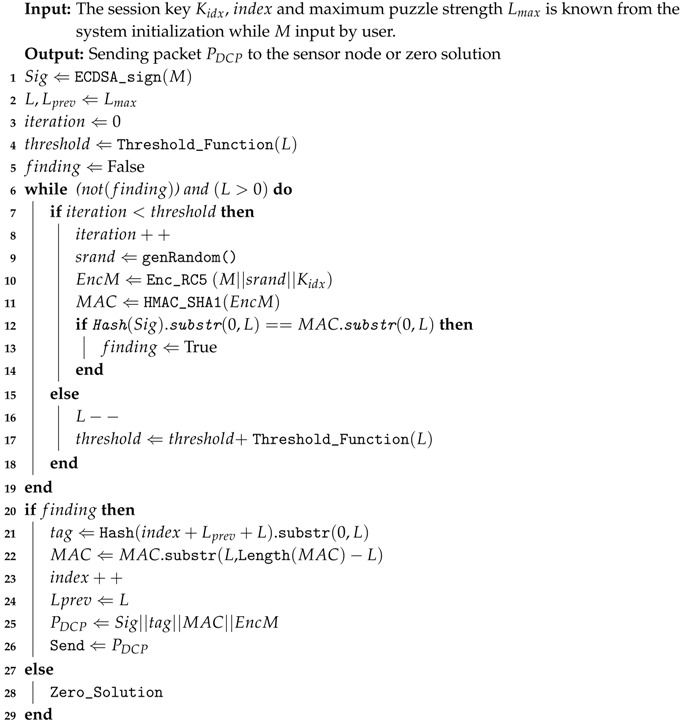



**Algorithm 2:** Dynamic Cipher Puzzle verification at the receiver-side

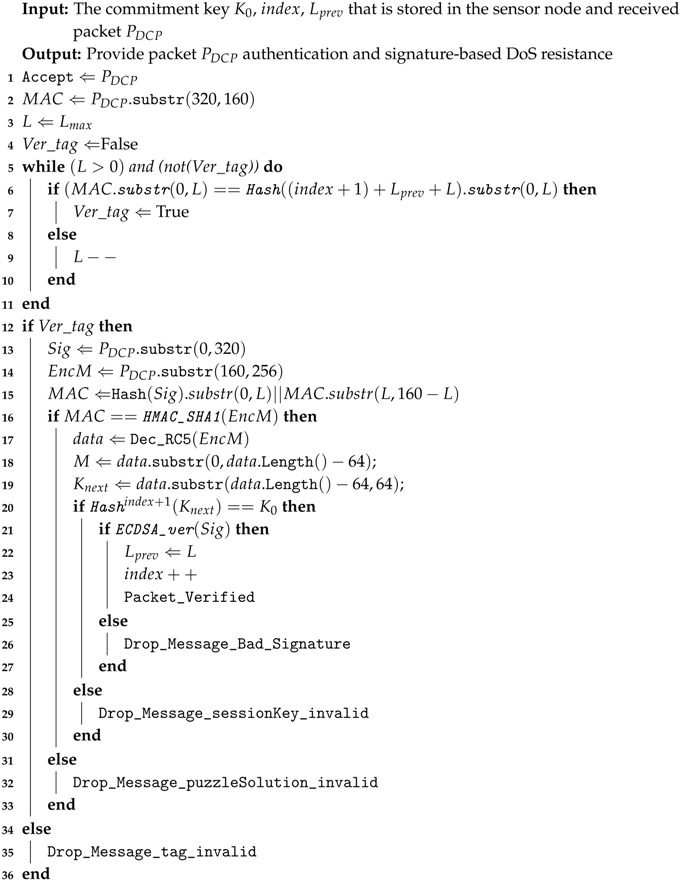



The initialization phase on the sender side is divided into two steps. Firstly, the sender has to determine the value of maximum puzzle strength ($L_{max}$), which contains the length of the pattern in units of bits. There is no exact value for this parameter. MSP recommends 22 bits [15], which is considered a proper number. The reason is that the higher is the puzzle strength, the higher is the number of trials needed to find the puzzle solution. Another motivation is the increase of sender-side delay. Secondly, the sender has to decide the length of the session key and build the keychain. There is no specific number for the key length, because re-keying methods can be used. The activity of the sender starts with digital signature creation. ECDSA, a PKC-based signing method, is used (Line 1 in Algorithm 1). Later, the threshold value is determined based on the maximum puzzle strength and the selected threshold function (Lines 2–4 in Algorithm 1). Next, puzzle solution construction can be started (Lines 6–19 in Algorithm 1). The first activity is comparing the number of iterations with the threshold value (Line 7 in Algorithm 1). If the number of iterations is under the threshold value, then the sender can generate a random number for a candidate puzzle solution (Line 9 in Algorithm 1). The next step is ETM. This method aggregates the message, a random number, and the session key (Lines 10–11 in Algorithm 1). The first *L* bits of the ETM result are compared with the hash of the signature (Line 12 in Algorithm 1). If there is a match, then the puzzle solution has been found. Otherwise, DCP starts another trial by using a different random value for srand. This trial process is continued until the puzzle solution is found or the number of trials exceeds the threshold value. If this happens, then the threshold value is re-computed by adding a new threshold value from a lower value of puzzle strength (Lines 16–17 in Algorithm 1). The result of puzzle solution construction is either puzzle “solution created” or “zero solution”. After the puzzle solution has been found, the next process is tag creation (Line 21 in Algorithm 1). The final activity is to send a concatenation of signature, tag, MAC, and encrypted message (Lines 25–26 in Algorithm 1).

Reception starts with the incoming $P_{DCP}$ packet. The first process is tag verification (Line 2 in Algorithm 2), trying every possible value of the current puzzle strength (*L*) starting with maximum puzzle strength. The receiver has to try up to $L_{max}$ times for each incoming packet based on its stored index and previous puzzle strength. If the tag is valid, then the process is continued with verification of the puzzle, including the MAC (Lines 13–16 in Algorithm 2). If the MAC and puzzle are valid, then the receiver can decrypt the incoming message (Line 17 in Algorithm 2). Based on part of the decrypted message, session key verification can be started (Line 20 in Algorithm 2). This aims to deny fake puzzle solutions. The last activity is ECDSA verification (Line 21 in Algorithm 2) to ensure the authenticity of the sender.

## 5. Implementation and Performance Evaluation

This section discusses the experimental testing of the proposed methods. The proposed threshold functions were compared with quartile threshold functions [18]. The candidates are listed in Table 4.

**Figure 8:**
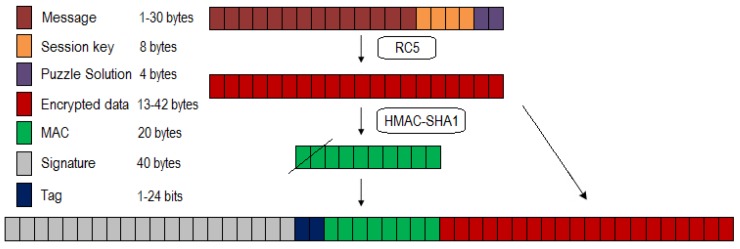
DCP packet content.

The first step after finding the threshold function candidates was sending a message from the administrator to the sensor nodes. Based on IEEE 802.15.4 standardization, the packet size was 102 bytes. In addition, the detailed payload for each sent packet in the DCP is shown in Figure 8. This consists of a digital signature, tag, MAC, and encrypted data that are composed of a message, session key, and puzzle solution. The message content may vary but the length is between 1 and 30 bytes, while the lengths of the session key and puzzle solution are 8 and 4 bytes, respectively. Consequently, the total length of the encrypted data is the sum of those values, its value ranging from 13 to 42 bytes. Furthermore, we used HMAC-SHA1 and ECDSA to produce 20 bytes of MAC and 40 bytes of signature. The maximum puzzle-strength is 24 bits. This value comes from a multiplication of 8 bits close to 22 bits, as recommended by Ning and Liu [15] considering system performance. Subsequently, the proposed schemes were simulated on Network Simulator-3 (NS-3) using the WSN module.

### 5.1. Simulation Results

**Figure 9:**
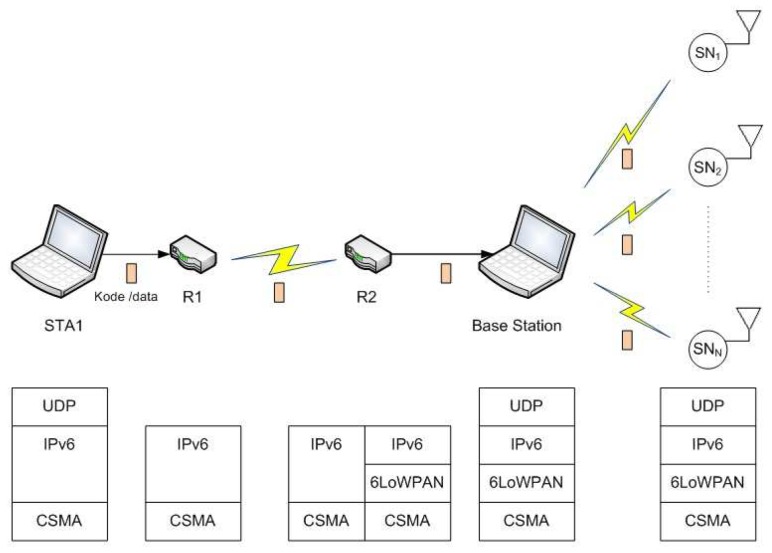
Network topology for the proposed scheme experiment using NS-3.

The detailed network topology of our proposed scheme experiment is illustrated in Figure 9. It consists of the sender, or the administrator (STA1), two routers (R1 and R2), one base station, and several sensor nodes ($SN_{1-N}$) as receivers.

Instead of data authentication, there is a further requirement to authenticate the entity or user. An administrator must be registered as a legitimate user. The main concern is not only the adversaries but also the resource constraint characteristics of wireless sensor networks. Smart-card-based password authentication by Wang and Wang [28] has been proposed. This provides security against the hardest adversary model. In addition, “fuzzy-verifier” and “honeywords” are integrated. This scheme avoids user, or sender, corruption and server, or receiver, compromised. Furthermore, it can be implemented in the single- or multi-server architecture. Moreover, mutual authentication protocol using Elliptic Curve Cryptography has been proposed [29] specific in the multi-server environments. This provides security against all known attacks, un-traceability, and perfect forward security. For simplicity, this paper does not take into account user authentication. The detailed mechanism of both user authentications explained in [28,29].

The simulation aims to explore threshold function behavior. Therefore, the proposed threshold functions were compared with quartile threshold functions [18] through four parameters: zero solution probability, mean number of hash iterations, standard deviation, and maximum number of hash iterations, as explained in Section 5.1.1, Section 5.1.2, Section 5.1.3 and Section 5.1.4, respectively.

#### 5.1.1. Zero Solution Probability

**Figure 10:**
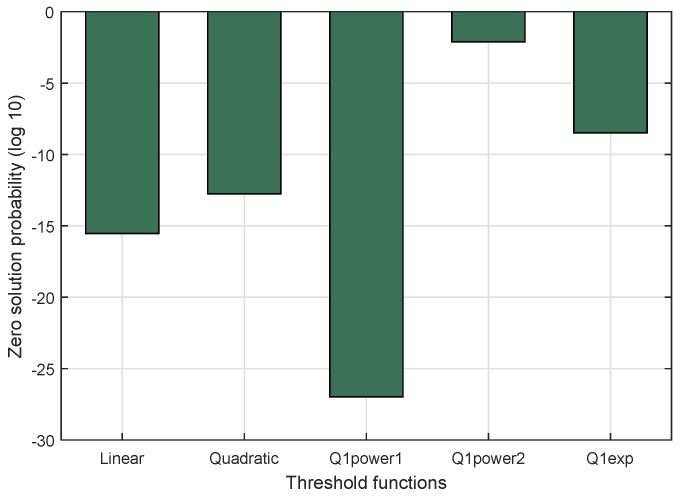
Zero solution probability (in log${}_{10}$) of threshold functions in DCP.

This parameter measures the probability of zero puzzle strength (the puzzle having no solution). This is crucial to the dynamic puzzle scheme, which focuses on reducing sender-side delay. As mentioned before, the system aims to get a puzzle solution in the first trial, ranging from $L_{max}$ to 1. This has an impact on the possibility of not finding a puzzle solution because the number of hash iterations is smaller than the average value. The proposed scheme, consisting of two possible threshold functions (Linear and quadratic), was compared with quartile threshold functions, i.e., Q1power1, Q1power2, and Q1exp [18]. The result is shown in Figure 10. It shows the logarithmic value of zero solution probability, because the deviation between them is high. The lowest value of zero solution probability was achieved by Q1power1 at 1.059 $\times 10^{-27}$ (−26.9 in log basis 10). The highest value of zero solution probability came from Q1power2 at 0.00766 (−2.1158 in log basis 10). The zero solution probability values of the linear and quadratic threshold functions were 2.961 $\times 10^{-16}$ (−15.5 in log basis 10) and 1.728 $\times 10^{-13}$ (−12.7 in log basis 10), respectively. They were lower than Q1exp but higher than Q1power1. This means that both functions performed better than Q1exp but worse than Q1power1.

#### 5.1.2. Mean Number of Hash Iterations

**Figure 11:**
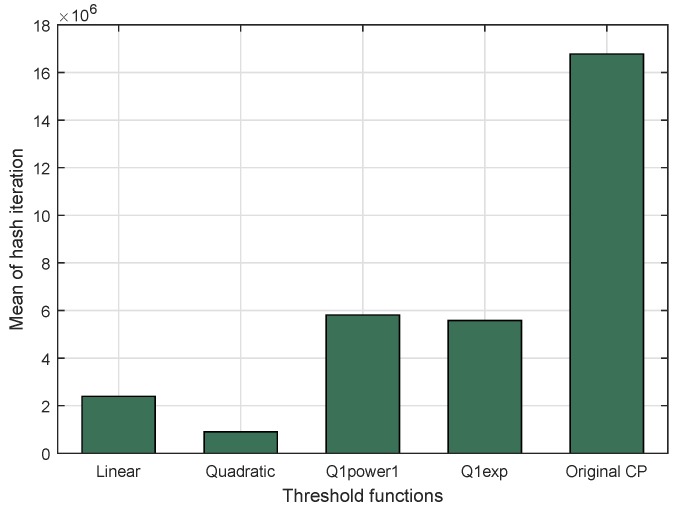
Mean number of hash iterations for DCP with four different threshold functions and original Cipher Puzzle.

The mean number of hash iterations was evaluated by repeating puzzle generation 500 times at the sender side. This experiment compared the use of DCP with four different threshold functions (Linear and quadratic for the proposed scheme; Q1power1 and Q1exp as quartile threshold functions [18]) and without threshold function [13]. The result is shown in the Figure 11. The highest value of the mean number of hash iterations definitely came from the original Cipher Puzzle without threshold function. This is because the system never stops until the solution is found; there is no boundary. Furthermore, the lowest value of the mean number of hash iterations was achieved by DCP using the quadratic threshold function. The value was 901,389 trials of the hashing process, i.e., a decrease by 94.6% compared to the mean number of hash iterations in the original Cipher Puzzle with 24-bit puzzle strength ($2^{24}$ = 16,777,216).

#### 5.1.3. Standard Deviation

**Figure 12:**
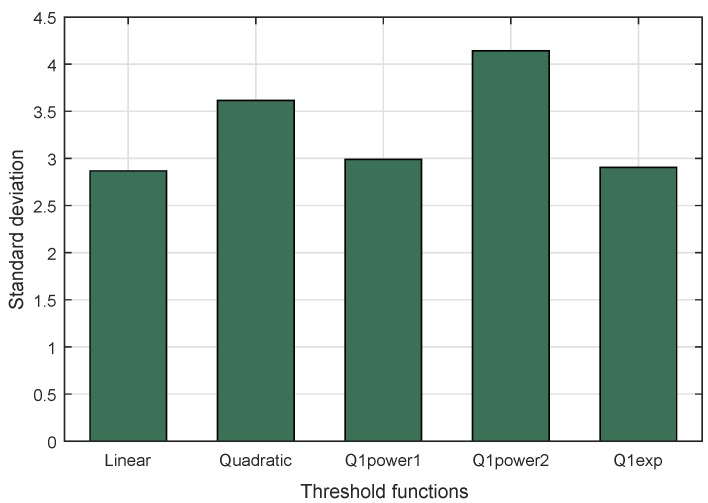
Standard deviation for threshold functions in DCP.

A high diversity of puzzle strength makes it difficult to guess by adversaries. This parameter was used to compare the four threshold functions when implemented in DCP. The result is shown in Figure 12. The highest standard deviation came from Q1power2, followed by Quadratic, Q1power1, and Q1exp, sequentially. The lowest standard deviation was produced by the linear threshold function.

#### 5.1.4. Maximum Number of Hash Iterations

**Figure 13:**
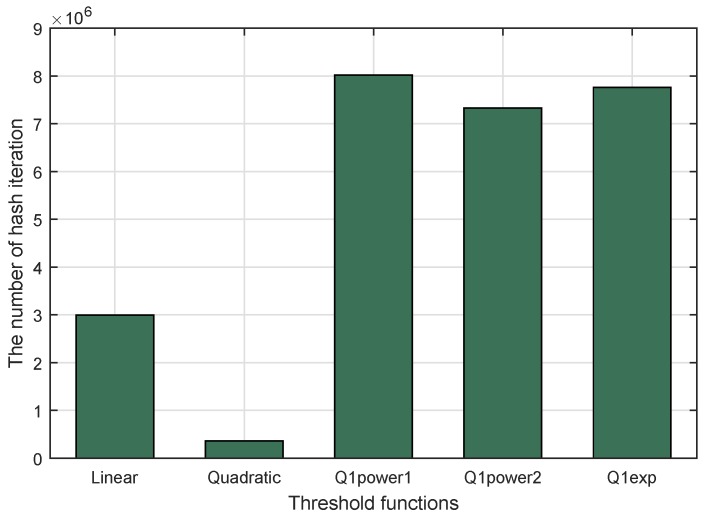
Maximum number of hash iterations for threshold functions in DCP.

This parameter was used to measure the maximum time or number of hash iterations needed for each puzzle generation. It compares the total number of hash iterations from the highest $L_{max}$ to the lowest puzzle strength (*L* = 1) from the proposed scheme (for linear and quadratic threshold functions) and the previous methods (for Q1power1, Q1power2, and Q1exp). The result is shown in Figure 13. The highest value of maximum number of hash iterations was achieved by Q1power1, followed by Q1exp, Q1power2, and the linear function, sequentially. The lowest value of maximum number of hash iterations came from the quadratic function. The deviation between the highest and the lowest value of the maximum number of hash iterations was 7,664,810. In other words, for the quadratic threshold function, it was lower by 95.54% than for Q1power1.

### 5.2. Experimental Results

**Figure 14:**
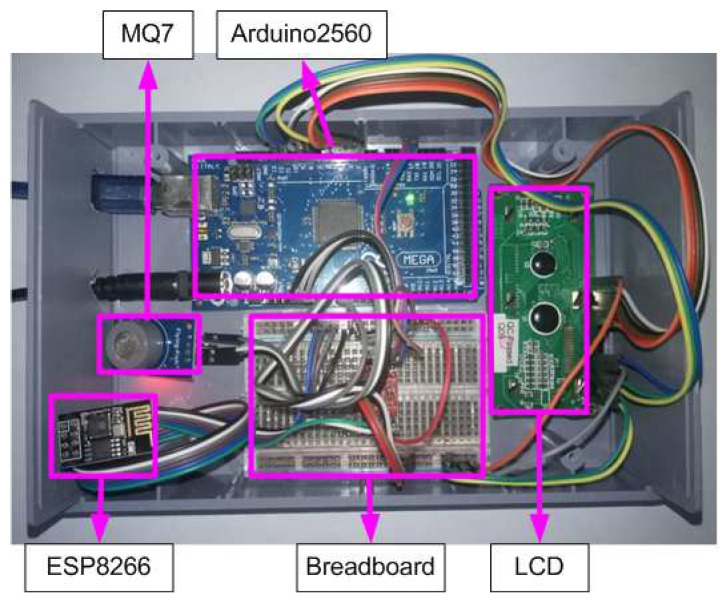
Photograph of the sensor node used in the experiment.

A simulation was made of a network construction for real communication between a sender and a wireless sensor network. The administrator utilized a workstation with specifications Intel Core i3-2310M CPU @ 2.10 GHz—4CPUs processor and 6144 MB DDR3. The receiver used an Arduino Mega 2560 microcontroller, consisting of a microcontroller, ESP8266 WiFi Shield, an MQ7 sensor, and an LCD. The details are shown in Figure 14. This node was used to detect poisonous gasses so the MQ7 was utilized to sense carbon monoxide (CO) concentrations in the air.

The main purpose of the experiment was to test the incoming packet verification process in the sensor nodes. The proposed method should decrease sender-side delay. Consequently, it increases the activity at the receiver-side. Therefore, the consumption of time, energy and other resources needed for each verification process was analyzed. The effect of tag verification from the dynamic puzzle in our proposed method (DCP) and DMP [18] was evaluated, as discussed in Section 5.2.1. In addition, the total resources needed for the full DCP verification process were observed based on four parameters: RAM, energy, and storage overhead. Finally, the DCP results were compared with those from Cipher Puzzle and DMP, as discussed in Section 5.2.2.

#### 5.2.1. Resource Consumption for Tag Verification

**Figure 15:**
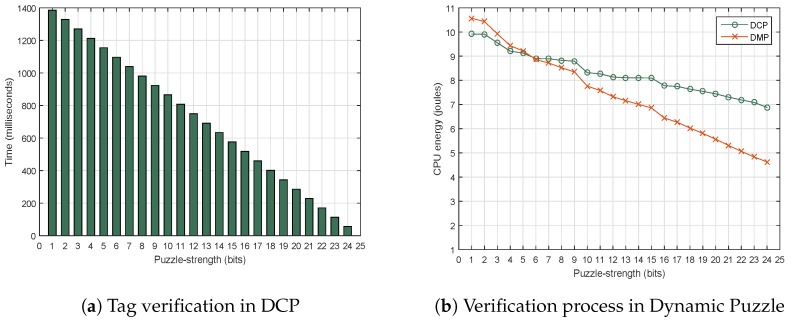
The resources of Dynamic Puzzle verification in Arduino.

Additional resources at the sensor node are required for tag verification. The receiver has to do up to $L_{max}$ trials of the hash function. The resources needed for each trial were analyzed, i.e., the time consumption and the CPU energy consumption for the dynamic puzzle, as illustrated in Figure 15. In Figure 15a, a lower puzzle strength sent by the administrator led to more energy and time needed by the sensor node for the verification process. The time interval for each trial was almost constant, i.e., about 57.749 milliseconds. This was the time needed for the concatenation and hash operations of each trial. Figure 15b illustrates a comparison of CPU energy consumption between DCP and DMP. The energy needed to verify tags was higher for DCP than for DMP at puzzle strength higher than 6-bit. However, the energy needed by DMP was higher than by DCP at puzzle strength lower than 6-bit, because the range of the energy interval for each puzzle strength increase in DMP was higher than in DCP. The reason is that bit manipulation in the tag of MSP is done on the whole received packet, while in the tag of DCP it is done on only the 20 bytes of the MAC.

#### 5.2.2. Resource Consumption for Full Verification

**Figure 16:**
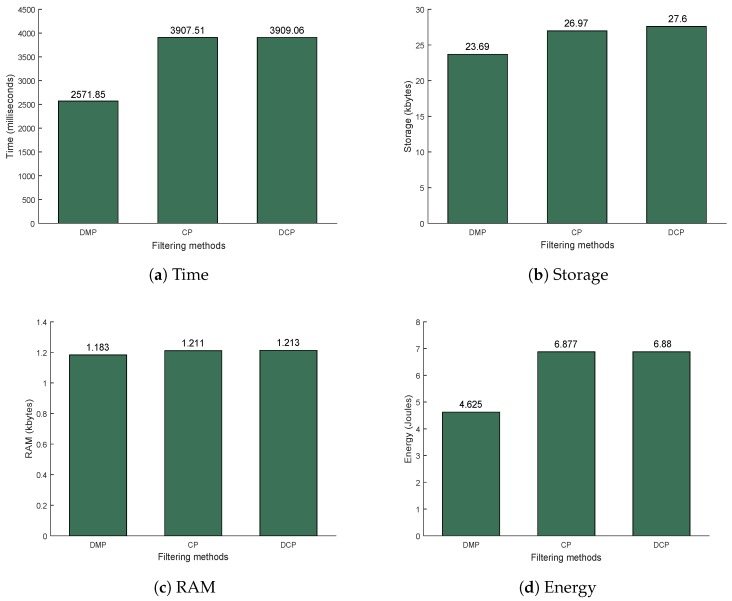
Resource consumption of DMP, CP and DCP verification processes in Arduino.

For each packet received, Arduino is required to run a series of verification processes to deal at least with signature-based DoS attacks. Three methods that utilize either dynamic (DMP and DCP) or static (Cipher Puzzle) puzzle strength were analyzed. The resources required by these three verification schemes can be seen in Figure 16. The resource consumption of DMP was the lowest. This is because DMP does not utilize a confidentiality mechanism, so there is no ETM activity in DMP; it only verifies tag, session key, puzzle, and signature while the other methods utilize ETM to guarantee confidentiality. DCP consumed the most resources. The value was higher than for Cipher Puzzle because, apart from ETM and signature verification, DCP also requires tag verification. This process is necessary to get information about the puzzle strength and the index of the sender. Even though DCP resource consumption was higher than that of Cipher Puzzle, the difference was quite small, i.e., only 0.0397%, 2.32%, 0.165% and 0.044% for time, storage, RAM, and energy, respectively. This is still acceptable. From the simulation results it can be seen that the quadratic threshold function in DCP outperformed the others. It got the best performance for three out of four parameters, i.e., mean, deviation, and maximum of number of hash iterations. This is because Q2power2 is not recommended for use as a threshold function due to the existence of zero solution puzzles [18]. Furthermore, zero solution probability for the quadratic function was not the lowest but still higher than for Q1power2 and Q1exp. This guarantees that the success probability of finding a solution is about $1-(10^{-10})$. Based on the experimental results, it can be seen that the energy needed for DCP verification was the highest. However, this additional energy is still acceptable compared with Cipher Puzzle. The additional resources are offset by decreased sender-side delay.

## 6. Security Analysis

This section gives a brief explanation of the puzzle security of the proposed scheme and how the proposed scheme faces attacks that are detailed explained using adversary model. Two types of attacks are discussed: probability attacks and signature-based DoS attacks.

### 6.1. Puzzle Security

There are three main properties for a secure puzzle protocol in order to resilience against resource exhaustion DoS attacks, i.e., unforgeability, difficulty preservation, and solver identity complexity [30]. The first property of DCP can be known by looking at its formal definition. The puzzle concept is the same as in other methods, such as HashTrail and HashInversion [30], but the details differ. The formal definition of the DCP scheme follows the idea from [30] and can be explained as follows:

Dynamic Cipher Puzzle Let $H:{\left\{0,1\right\}}^{*}\to {\left\{0,1\right\}}^{k}$ be a publicly known hash function and a fix $LSpace=[1,L_{max}]$, $pSpace={\left\{0,1\right\}}^{*}\times {\left\{0,1\right\}}^{k}$ and $sSpace={\left\{0,1\right\}}^{*}$. The Dynamic Cipher Puzzle is a quadruple of algorithms:

- Setup $\left(1^{k}\right)$ is the setup algorithm that on input $1^{k}$ outputs **atr** $=K_{idx+1}$,
- Gen (L) is the generation algorithm which on input *L* randomly chosen $x\in {\left\{0,1\right\}}^{k}$, computes H(Sig(x)) and outputs puzzle instance **puz** $=\{L,x,H\left(Sig\left(x\right)\right),K_{idx+1}\}$
- Find (**puz**$,t_{max})$ is the solving algorithm that on input **puz** and the number of steps $t_{max}$ iteratively samples at most $t_{max}$ values **sol** $\in {\left\{0,1\right\}}^{L}$ until **ETM**${}_{k_{idx}}(x∥$**sol**$∥K_{idx+1}{)}_{1..L}=H{\left(Sig\left(x\right)\right)}_{1..L}$
- Ver(**puz**, **sol**) is the algorithm that takes **puz**, **sol** as input and return one if **ETM**${}_{k_{idx}}(x\;∥$**sol**$∥K_{idx+1}{)}_{1..L}=H{\left(Sig\left(x\right)\right)}_{1..L}$ and zero otherwise.

Based on DCP’s formal definition, it can be concluded that this scheme is unforgeable. It assures DoS resilience by combining a puzzle with MAC using a session key. Thus, it is hard for adversaries to find the solution without knowing the session key and index that match the puzzle strength (also called puzzle difficulty).

The second property is difficulty preservation. It consists of fairness of the first kind, or fairness in answering, and fairness of the second kind, or fairness in solving [30]. Fairness in answering means that if the adversary fails to solve the puzzle then it is bounded by *t* iterations. The bound is negligible and constant in the puzzle strength parameter. The average iterations needed to produce an *L*-bit puzzle is stated in Equation (13). If an adversary fails to find the solution, then he may exceed the minimum iterations value. This means DCP has fairness in answering. Fairness in solving means that an adversary cannot produce a puzzle solution in fewer than $t_{max}$ iterations bounded by negligible and constant puzzle strength. DCP holds this property since, regardless of the puzzle strength value, the number of $t_{max}$, stated in Equation (5), is constant.

**Figure 17:**
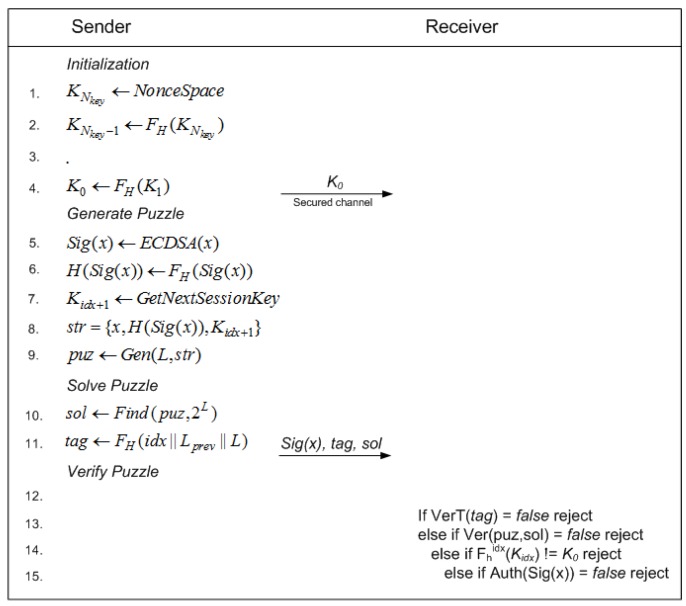
DCP protocol.

The third property of DCP can be known by looking at the protocol details of the DCP scheme, as shown in Figure 17. Puzzle generation and solving is initiated by the sender. Therefore, an adversary does not have any information about the puzzle’s content. Furthermore, the identity of the solver in the DCP scheme can be known through its signature. This study selected ECDSA, which has a 20-byte length for both the public key and the private key. This is safe enough to authenticate the identity of the sender.

### 6.2. Adversary Model

This section discusses the details of the environment of the adversary. It aims to make an assessment of the security model. Furthermore, an adversary model is developed to represent a real threat. This model was inspired by previous works [29,31]. The capabilities of adversary A are:

C-1. A is able to control of the exchange message between sender or administrator, base station and sensor nodes.
C-2. A can enlist all the combination item of tag $D_{idx}\times D_{L}\times D_{Lprev}$ within polynomial time, where $D_{idx}$, $D_{L}$ and $D_{Lprev}$ refer to the space of index, current puzzle-strength (*L*) and previous puzzle-strength($L_{prev}$), respectively.
C-3. A can compromise and reveal parameters stored in several sensor nodes.
C-4. A can create and transmit fake puzzle as if a legitimate user.

Capability C-1 means that A can eavesdrop, intercept, delete, modify or resend a transmitted message. Capability C-2 implies that the space of each item in the tag is limited. The puzzle solution length is 4 bytes. This value was chosen in order to reduce space overhead. The maximum puzzle strength for this condition that has been tested is 26-bit [15]. Therefore, the puzzle strength range that can be used is [1..26]. Meanwhile, index represents the position of the session key. Therefore, the maximum value of index is Nkey. The size of the tag is limited by [Nkey×26×26]. Capability C-3 is common, because the sensor nodes are usually placed in hostile areas and receive minimum attention. The last capability, C-4, refers to A trying to produce a large copy of the puzzle with lower puzzle strength. This kind of attack aims to deplete the resources of the sensor node.

#### 6.2.1. Probability Attack

The principle of a probability attack is finding a fake combination of puzzle and solution with the same puzzle strength as that of the system [16]. This attack has the same possibility as the system to find the puzzle solution. It can be mitigated by using a dynamic length for puzzle strength and pattern. Both parameters have different values for each packet that is sent. Based on capabilities C-1 and C-2, A can intercept transmitted message [Sig,tag,MAC,EncM]. Therefore, A can find a fake puzzle solution as follows:

Step 1: Pick a combination $\left(idx_{i}^{*},L_{i}^{*},Lprev_{i}^{*}\right)$ from dictionary $D_{idx}$, $D_{L}$ and $D_{Lprev}$.
Step 2: Compute $tag_{i}^{*}=F_{h}\left(idx_{i}^{*}+L_{i}^{*}+Lprev_{i}^{*}\right)$.
Step 3: Validate correctness of $tag_{i}^{*}$ by checking if the first $L_{i}^{*}$ bits of the $tag_{i}^{*}$ and the intercepted tag are the same.
Step 4: Repeat Steps 1–3 until the correct combination is found to form a valid tag.
Step 5: Calculate $MAC_{i}^{*}$ from the hash result of the concatenation of the first $L_{i}^{*}$ bits of the intercepted Sig and the first ($160-L_{i}^{*}$) bits of the rest intercepted message after $tag_{i}^{*}$.
Step 6: Calculate the predicted position of encrypted message, session key and puzzle solution as $EncM_{i}^{*}$ in the rest of the intercepted message after $MAC_{i}^{*}$.
Step 7: Validate correctness of the puzzle solution by checking if the ETM of the $EncM_{i}^{*}$ using the last stored session key $Kidx_{i}^{*}$ and the $MAC_{i}^{*}$ are the same.

Steps 1–3 in the above activity aim to get information about the index and transmitted puzzle strength. It appears to be a simple method but collision cannot be avoided. The tag value is added among three variables that are in almost the same range. Assuming that the number of keys is $10^{2}$, the number of possible combinations of tags is 100×26×26= 67,600 items. However, a valid combination cannot be used directly. The predicted tag value may be wrong, so A must ensure that the puzzle is valid by continuing to Step 7. Steps 5 and 6 determine the position of the intercepted MAC and EncM. If the predicted tag is invalid, then the predicted MAC and EncM are invalid too. Before continuing to the last step, A has to predict the session key. The length of the key is 64 bits. Using a brute force method, A has to try $2^{64}$ combinations. Consequently, the obscurity depends on the session key as the center of randomness in the MAC production. The time constraint of this type of attack is determined by the time interval $T_{interval}$ of the transmitted packet. It needs only 6.53 ms (payload 102 bytes and bandwidth 250 kbps) [13].

#### 6.2.2. Signature-Based DoS Attacks

Signature-based DoS attacks are broadcast attacks that utilize a fake signature to flood communication in the sensor node. Signature verification is an expensive computation in resource-constrained devices; it can still be implemented but only limitedly. In addition, signature-based DoS attacks disturb the communication in the sensor node. To succeed in sending a fake signature, A must pass the tag and DCP verification. The details of fake puzzle generation are described in Section 6.2.1. If A passes Step 7, then the predicted session key must be validated before the main authentication verification. There are two conditions in which A can pass Step 7. Firstly, the session key is valid and the encrypted puzzle solution is valid too. Secondly, the session key looks valid (but in fact is invalid) because the encrypted puzzle solution is invalid. The second condition can occur because the predicted combination of encrypted puzzle solution and session key is wrong. If the first condition occurs, then A can directly attack the main signature by guessing the public key of the sender. This is because the list of authorized public keys is stored in the sensor node. If A has capability C-3 then he will also have C-4, which means that the session key and authorized public keys are exposed. Therefore, the compromised node must be detected and exiled from the system, for which several optional mechanisms can be used [32,33,34]. For simplicity, the details of these mechanisms are not discussed in this manuscript. Moreover, the sender or administrator must initiate re-keying of the session key. It will be broadcast along with the list of authorized public keys to the non-compromised sensor nodes through a secure channel.

## 7. Conclusions

In this research, the performance of a new filtering method added to Cipher Puzzle, specifically for wireless sensor networks, was investigated. This method aims to mitigate signature-based DoS attacks. In the implementation of Cipher Puzzle, there is an uncontrollable event in finding the puzzle solution that creates sender-side delay. This paper proposes the Dynamic Cipher Puzzle (DCP) scheme, which uses linear and quadratic threshold functions to reduce sender-side delay, so the sender can process the outgoing packets more efficiently. The simulation results show that DCP using a quadratic threshold function outperformed the other methods, decreasing sender-side delay by 94% with the guarantee of finding a puzzle solution. Furthermore, the high standard deviation and implicit puzzle strength make the attack complexity higher than using static puzzle strength.

As a result of additional activity, the resources used by the sensor nodes increase. Thus, the consumption of time, storage, RAM and energy on the receiver-side goes up but is still acceptable, i.e., the increase is under 3%. In the future, it is desirable to develop a scheme for multi-hop wireless sensor networks that increases the experimental challenge.

## Figures and Tables

**Table 1:** Nomenclature.

Notation	Description
nH	Hash iteration
$\bar{nH}$	Mean of hash iteration
n(M)	Length of message
index	Number of packet transmissions
Sig	Signature
$K_{idx}$	Session key for index idx
$P_{idx}$	Puzzle solution for index idx
*L*	Puzzle-strength
$L_{max}$	Maximum value of puzzle-strength
$N_{key}$	Number of keys
$F_{N}$	Hash Function
$PS_{\left[success,i\right]}$	Success probability in the puzzle-strength equal to *i*
$P_{success}$	Success probability of the system
$P_{failure}$	Failure probability of the system
$P_{ZS}$	Zero solution probability
$n_{DCP}$	Number of hash iteration in the DCP
$\delta_{L}$	Standard deviation of the puzzle-strength set

**Table 2:** Comparison between Client Puzzle, Message Specific Puzzle and Cipher Puzzle.

No	Description	Client Puzzle [14]	MSP [15]	Cipher Puzzle [13]
1	The role of parties in puzzle generation	Server creates and verifies the puzzle; Client solves the puzzle from the server.	Sender creates and solves the puzzle; Receiver verifies the puzzle.	Sender creates and solves the puzzle; Receiver verifies the puzzle.
2	Security property guarantee	Authentication based on puzzle and main signature.	Authentication based on puzzle and main signature.	Authentication based on puzzle and main signature; Confidentiality based on encryption of the message content.
3	Pattern of the puzzle solution	Consecutive zero bits.	Consecutive zero bits.	Hash result of the message’s signature.
4	Procedure composition	Hash function and main signature (plaintext packet content).	Hash function and main signature (plaintext packet content).	Hash function, encrypt-then-MAC to provide high security level on the transmitted packet, and main signature.
5	Session key	-	One-way key chain.	One-way key chain.

**Table 3:** Success Probability Summary for Optimum Threshold Function Selection.

Area	Success Probability on Puzzle Strength		Parameter	
	*L* = 1	*L* = 2	Optimum	Worst
I	LOW	LOW	$n_{DCP}$, $\sigma_{L}$	$P_{ZS}$
II	HIGH	LOW		
III	LOW	HIGH		
IV	HIGH	HIGH	$P_{ZS}$	$n_{DCP}$, $\sigma_{L}$

**Table 4:** Threshold functions.

Threshold Function Name	Description
Linear	$\left\lceil \frac{\log_{10}\left\{1-\left[\left(1-10^{-10}\right)\frac{\left[L-\left(L_{max}+1\right)\right]}{\left[-L_{max}\right]}\right]\right\}}{\log_{10}\left\{1-{\left(\frac{1}{2}\right)}^{L}\right\}}\right\rceil$
Quadratic	$\left\lceil \frac{\log_{10}\left\{1-\left[\frac{\left(1-10^{-10}\right)}{{L_{max}}^{2}}{\left(L-\left(L_{max}+1\right)\right)}^{2}\right]\right\}}{\log_{10}\left\{1-{\left(\frac{1}{2}\right)}^{L}\right\}}\right\rceil$
Q1power1 [18]	$\left\lceil 1.8786^{L}+1726\right\rceil$
Q1power2 [18]	$\left\lceil L^{12.4}\times \left(2.412e-11\right)\right\rceil$
Q1exp [18]	$\left\lceil e^{L\times 0.5997}\times 1.966\right\rceil$

## References

[1] Eldefrawy M.H., Khan M.K., Alghathbar K., Cho E.S. (2010). Broadcast Authentication for Wireless Sensor Networks Using Nested Hashing and the Chinese Remainder Theorem. Sensors.

[2] Zheng X.L., Wan M. (2014). A Survey on Data Dissemination in Wireless Sensor Networks. J. Comput. Sci. Technol..

[3] Grover K., Lim A. (2015). A survey of broadcast authentication schemes for wireless networks. Ad Hoc Netw..

[4] Perrig A., Szewczyk R., Tygar J.D., Wen V., Culler D.E. (2002). SPINS: Security protocols for sensor networks. Wirel. Netw..

[5] Liu D., Ning P. (2004). Multilevel *μ* TESLA: Broadcast Authentication for Distributed Sensor Networks. ACM Trans. Embed. Comput. Syst..

[6] Groza B. (2008). Broadcast authentication with practically unbounded one-way chains. J. Softw..

[7] Kwon T., Hong J. (2010). Secure and efficient broadcast authentication in wireless sensor networks. IEEE Trans. Comput..

[8] Liu A., Ning P. TinyECC: A Configurable Library for Elliptic Curve Cryptography in Wireless Sensor Networks. Proceedings of the 7th International Conference on Information Processing in Sensor Networks.

[9] Ren K., Yu S., Lou W., Zhang Y. (2009). Multi-user broadcast authentication in wireless sensor networks. IEEE Trans. Veh. Technol..

[10] Liu Y., Li J., Guizani M. (2012). PKC based broadcast authentication using signature amortization for WSNs. IEEE Trans. Wirel. Commun..

[11] Cheng C.Y., Lin I.C., Huang S.Y. (2015). An RSA-Like Scheme for Multiuser Broadcast Authentication in Wireless Sensor Networks. Int. J. Distrib. Sens. Netw..

[12] Dong Q., Liu D., Ning P. (2013). Providing DoS resistance for signature-based broadcast authentication in sensor networks. ACM Trans. Embed. Comput. Syst..

[13] Tan H., Ostry D., Zic J., Jha S. (2013). A confidential and DoS-resistant multi-hop code dissemination protocol for wireless sensor networks. Comput. Secur..

[14] Aura T., Nikander P., Leiwo J. (2000). DOS-resistant authentication with client puzzles. International Workshop on Security Protocols.

[15] Ning P., Liu A.N. (2008). Mitigating DoS Attacks against Broadcast Authentication in Wireless Sensor Networks. ACM Trans. Sens. Netw..

[16] Du X., Chen H.H. Defending DoS Attacks on Broadcast Authentication in Wireless Sensor Networks. Proceedings of the 2008 IEEE International Conference on Communications.

[17] Dong Q., Liu D. Pre-Authentication Filters: Providing DoS Resistance for Signature-Based Broadcast Authentication in Sensor Networks. Proceedings of the First ACM Conference on Wireless Network Security.

[18] Afianti F., Wirawan I., Suryani T. (2018). Dynamic Message Puzzle as Pre-Authentication Scheme in Wireless Sensor Networks. Int. J. Adv. Sci. Eng. Inf. Technol..

[19] Wijaya D.R., Sarno R., Zulaika E., Sabila S.I. (2017). Development of mobile electronic nose for beef quality monitoring. Procedia Comput. Sci..

[20] Lamport L. (1981). Password authentication with insecure communication. Commun. ACM.

[21] Afianti F., Wirawan I., Suryani T. Filtering methods for broadcast authentication against PKC-based denial of service in WSN: A survey. Proceedings of the Fifth International Conference on Wireless and Optical Communications.

[22] Meulenaer G.D., Gosset F., Standaert F.X., Pereira O. On the Energy Cost of Communication and Cryptography in Wireless Sensor Networks. Proceedings of the IEEE International Conference on Wireless and Mobile Computing (WIMOB’08).

[23] Sethi M., Arkko J., Keranen A. End-to-end Security for Sleepy Smart Object Networks. Proceedings of the IEEE 37th Conference onLocal Computer Networks Workshops (LCN Workshops).

[24] Xu A., Li M., Cai J., Xue N., Zhang J., Liu D., Craig P., Huang X. Improving Efficiency of Authenticated OpenFlow Handshake using Coprocessors. Proceedings of the IEEE 8th International Conference on Information Technology in Medicine and Education (ITME).

[25] Johnson D., Menezes A., Vanstone S. (2001). The Elliptic Curve Digital Signature Algorithm (ECDSA). Int. J. Inf. Secur..

[26] Cao X., Kou W., Dang L., Zhao B. (2008). IMBAS: Identity-based multi-user broadcast authentication in wireless sensor networks. Comput. Commun..

[27] Jech T. (2013). Set Theory.

[28] Wang D., Wang P. (2018). Two Birds with One Stone: Two-Factor Authentication with Security beyond Conventional Bound. IEEE Trans. Dependable Secur. Comput..

[29] Xu G., Qiu S., Ahmad H., Xu G., Guo Y., Zhang M., Xu H. (2018). A multi-server two-factor authentication scheme with un-traceability using elliptic curve cryptography. Sensors.

[30] Groza B., Warinschi B. (2014). Cryptographic puzzles and DoS resilience, revisited. Des. Codes Cryptogr..

[31] Wang D., Li W., Wang P. (2018). Measuring Two-Factor Authentication Schemes for Real-Time Data Access in Industrial Wireless Sensor Networks. IEEE Trans. Ind. Inform..

[32] Jan M.A., Nanda P., He X., Liu R.P. A Sybil Attack Detection Scheme for a Centralized Clustering-based Hierarchical Network. Proceedings of the Trustcom/BigDataSE/ISPA.

[33] Zheng Z., Liu A., Cai L.X., Chen Z., Shen X.S. (2016). Energy and Memory Efficient Clone Detection in Wireless Sensor Networks. IEEE Trans. Mob. Comput..

[34] Al-Riyami A., Zhang N., Keane J. (2016). An Adaptive Early Node Compromise Detection Scheme for Hierarchical WSNs. IEEE Access.

